# Adult Age Differences in Using Information From the Eyes and Mouth to Make Decisions About Others’ Emotions

**DOI:** 10.1093/geronb/gbac097

**Published:** 2022-08-10

**Authors:** Gillian Slessor, Pauline Insch, Isla Donaldson, Vestina Sciaponaite, Malgorzata Adamowicz, Louise H Phillips

**Affiliations:** School of Psychology, University of Aberdeen, Aberdeen, UK; School of Psychology, University of Aberdeen, Aberdeen, UK; School of Psychology, University of Aberdeen, Aberdeen, UK; School of Psychology, University of Aberdeen, Aberdeen, UK; School of Psychology, University of Aberdeen, Aberdeen, UK; School of Psychology, University of Aberdeen, Aberdeen, UK

**Keywords:** Aging, Emotion perception, Social cognition, Social interaction

## Abstract

**Objectives:**

Older adults are often less accurate than younger counterparts at identifying emotions such as anger, sadness, and fear from faces. They also look less at the eyes and more at the mouth during emotion perception. The current studies advance understanding of the nature of these age effects on emotional processing.

**Methods:**

Younger and older participants identified emotions from pictures of eyes or mouths (Experiment 1) and incongruent mouth–eyes emotion combinations (Experiment 2). In Experiment 3, participants categorized emotions from pictures in which face masks covered the mouth region.

**Results:**

Older adults were worse than young at identifying anger and sadness from eyes, but better at identifying the same emotions from the mouth region (Experiment 1) and they were more likely than young to use information from the mouth to classify anger, fear, and disgust (Experiment 2). In Experiment 3, face masks impaired perception of anger, sadness, and fear more for older compared to younger adults.

**Discussion:**

These studies indicate that older people are more able than young to interpret emotional information from the mouth, they are more biased to use information from the mouth, and suffer more difficulty in emotion perception when the mouth is covered with a face mask. This has implications for social communication in different age groups.

Accurate and efficient perception of facial expressions of emotion is a fundamental social skill (Montagne et al., 2007). Numerous studies have reported evidence of adult age-related impairments in perceiving emotions such as anger, sadness, and fear (summarized recently in Hayes et al., 2020). There is evidence that older adults also carry out emotion perception tasks using a different attentional strategy compared to younger: older adults focus less on eyes and more on mouths (see Grainger & Henry, 2020, for a meta-analysis). In the current studies, we investigate in more detail age differences in using information from the eyes and the mouth to make emotional decisions.

Previous eye-tracking studies found that older adults fixated less on the upper half of the face compared to young during emotion perception (e.g., Wong et al., 2005), and fixated proportionately less on the eye region than younger adults and instead looked more to the mouth (e.g., Murphy & Isaacowitz, 2009; Sullivan et al., 2007, though see Ebner et al., 2011). Grainger and Henry (2020) show that these age differences in attending to the eyes and mouth are reliable across 11 published studies.

An important factor to consider is that emotions may differ in the extent to which information from the eye and mouth regions is diagnostic. The relative importance of the eyes and mouth depends on which emotion is being displayed (e.g., Calder et al., 2000; Wegrzyn et al., 2017) and this may be due to differences in the muscle patterns used when expressing these emotions. Classifying disgust and happiness is most dependent on information from the lower half of the face, while classifying sadness, anger, and fear relies more on the information from the upper face (Wegrzyn et al., 2017). Older adults have particular difficulties in perceiving angry, fearful, and sad facial expressions (Hayes et al., 2020), so age-related declines in perception of these emotional expressions may be due to older adults avoiding looking at the eyes, which are critical for distinguishing these emotions (Murphy & Isaacowitz, 2009; Sullivan et al., 2007). It has also been claimed that the relative preservation in labeling disgusted and happy facial expressions among older adults may reflect the importance of the mouth region in these emotions (Sullivan et al., 2007).

Wong et al. (2005) found that both younger and older adults who made more fixations to the top half of angry, fearful, and sad faces were better at perceiving these emotions. However, subsequent studies found no significant correlations between the duration or number of fixations to the eyes/mouths and emotion perception performance (Grainger et al., 2017; Murphy & Isaacowitz, 2009). This might be because a shorter time looking at the eyes could indicate efficient processing of emotions or instead a maladaptive strategy of avoiding looking at this region. Also, Birmingham et al. (2018) point out that these eye-tracking studies only tell us about foveal overt attention over extended time periods, whereas the crucial processes for emotion perception may plausibly be rapid, covert, and sometimes parafoveal.

Older adults may focus more on the mouth region because they are better at decoding mouth-related cues compared to those from the eyes. This might be because life-span changes in hearing result in increasing levels of attention being focused on others’ mouths (Chaby et al., 2017; Wong et al., 2005), or because there are generational changes in social etiquette regarding eye contact, or because age-related changes in visual perception affect more on the more subtle cues from the eye region. However, there is limited available evidence to evaluate whether there are age differences in the ability to decode emotions from the eyes and mouth. The main aim of the current research is to investigate whether there are age-related differences in the *ability* to categorize emotions when the eye and mouth region are presented alone (Experiment 1), and also to directly measure whether older adults are more *biased* to use information from the mouth when making decisions about incongruent emotional faces (Experiment 2). We finally explore the potential *implications* of these age changes for everyday interactions during the coronavirus (COVID-19) pandemic by testing whether face masks particularly impair emotion perception for older adults (Experiment 3).

## Experiment 1: Age Differences in Identifying Emotions From Eye and Mouth Regions Alone

If older adults’ tendency to attend less to the eyes reflects poorer ability to actually decode information about this facial region, older adults should be less accurate than younger when decoding information from eyes alone. Also, older adults may retain greater ability to decode emotional information from the mouth region because they use this information more in everyday interactions. Age-related difficulties in identifying emotions from the eyes are likely to be strongest for sadness, anger, and fear, which have generally been associated with processing information from the eye region (Calder et al., 2000).

A previous study (Sullivan et al, 2007; Experiment 1) presented participants with photographs of only the eyes or only the mouth region expressing the six “basic” emotions. Their analysis suggested that younger adults show an advantage for perceiving emotions from the eyes compared to the mouth which was not seen in older people. However, they did not make a direct age comparison in their analysis. Also, for each emotion condition only two faces were presented at 100% emotional intensity, so there was limited variance in performance. In the current study, we included a larger number of stimuli of varying levels of intensity in order to more directly test whether age differences in emotion perception were greater for viewing eyes alone compared to mouths alone.

In the current study, we directly tested the hypothesis that age differences in emotion perception would be greater when only viewing the eyes compared to only viewing the mouth region of the face. We based this hypothesis on the evidence that older adults are less likely to look at the eyes and more likely to look at the mouth when viewing facial expressions (Grainger & Henry, 2020). We also tested whether this was particularly the case for the emotions of sadness, anger, and fear.

### Method

#### Power analysis

A statistical power analysis was performed for sample size estimation, based on data from Grainger and Henry (2020), who report a meta-analysis of the effects of age on looking at different face regions across eight studies. They report a mean effect size of *g* = 0.66 in terms of age differences in tendency to look at the eye region of the face. We therefore calculated the projected sample size needed to test our hypothesis of an interaction between age group (2: young, old) and face region (3: eyes, mouth, whole face) equivalent to an effect size of *f* = 0.33. With alpha = 0.05 and power = 0.95 (G*Power 3.1, analysis of variance [ANOVA] repeated measures, within–between interaction, assuming correlation between different conditions of 0.3) suggests a minimum total sample size of *n* = 36. Our actual sample of *n* = 48 should therefore be sufficient to detect the hypothesized interaction. A G*Power sensitivity analysis with *n* = 48 revealed 95% power to detect a medium (*f* = 0.28) between–within interaction.

#### Participants

Two groups of participants completed the whole study: 25 younger adults (five males) aged 18–40 (*M* = 21.68, *SD* = 5.10) and 23 older adults (five males) aged 64–84 (*M* = 72.83, *SD* = 5.80). The younger group was recruited from the University of Aberdeen psychology department in return for course credit; older adults were recruited from the psychology participant panel and were reimbursed for their time. All participants were free from reported neuropsychological disorders and had normal or corrected to normal vision. Those who required corrective lenses wore them during the experiment.

#### Stimuli

Photographs of two actors (one male, one female) displaying each of the six basic emotions (anger, fear, sadness, happiness, surprise, and disgust) at three levels of emotional intensity (50%, 75%, and 100%) were taken from the Facial Expressions of Emotion: Stimuli and Test (FEEST; Young et al., 2002). Lower intensity emotions were included to increase variability and avoid ceiling effects. In each image (11 cm × 14 cm), the actor’s hair was masked, so only the face was visible. The eye region and mouth region of the face in each image were isolated and cropped to 9 cm × 3 cm. Each of the eye-only images was cropped just above the eyebrows and below the bridge of the nose. The mouth-only images were cropped below the nose and above the chin so that only the mouth was visible.

#### Procedure

There were three different region conditions; whole face, eyes only, and mouth only. In each condition, images were presented individually in the center of a computer screen in a randomized order. Participants were asked to decide which of the six basic emotions was being portrayed and say their response aloud for the experimenter to record. The list of six possible emotion responses was presented in front of the laptop and each image remained on screen until the participant had made their decision. There were 36 trials in each condition. The order of the conditions was counterbalanced across participants.

### Results and Discussion

To assess whether region and emotion influenced age-related differences in emotion perception, a 3 (region: face, eyes, mouth) × 6 (emotion: anger, disgust, fear, happy, sad, and surprise) × 2 (age group: young, old) mixed-design ANOVA was used. The dependent variable was percentage correct. In all analyses in this paper, where assumptions of sphericity were violated, Huynh–Feldt-corrected *p*-values are reported. Also, we use planned Bonferroni-corrected pairwise comparisons to test for age differences in each condition.

**Figure 1.**
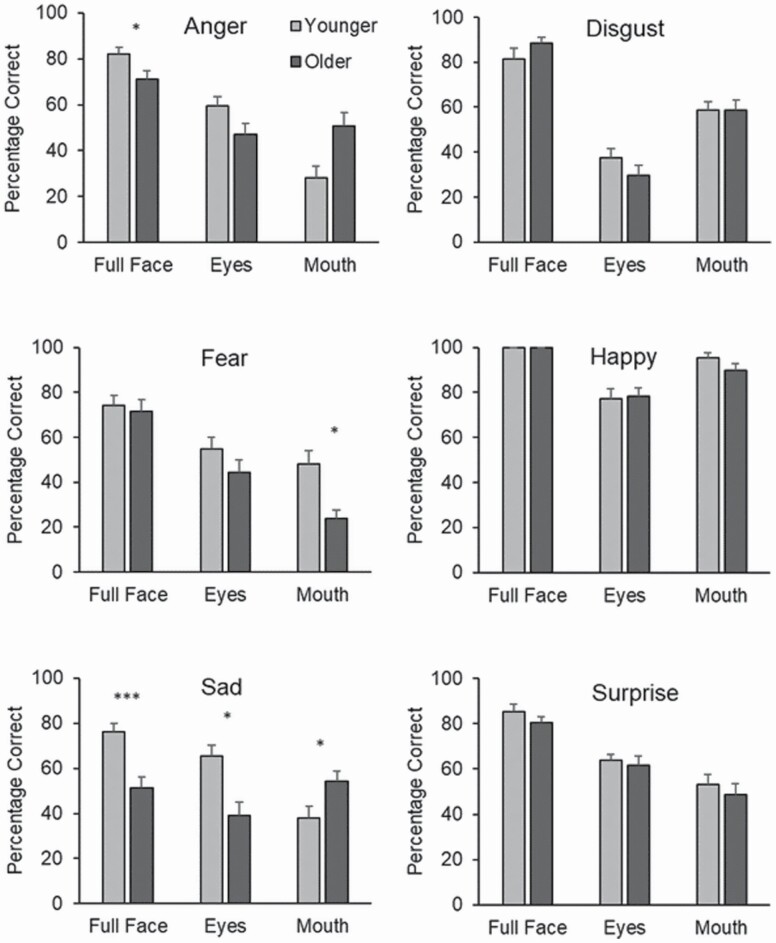
Experiment 1, mean percentage correct for each emotion category broken down by region and age group. Error bars denote standard error. Throughout, asterisks denote age differences at *p <.05, **p <.01, ***p < .001.

Significant main effects were found for region, *F*(2, 92) = 197.95, *p* < .001, η _*p*_^2^ = 0.81, emotion *F*(5, 230) = 51.16, *p* < .001, η _*p*_^2^ = 0.52, and age group, *F*(1, 46) = 5.93, *p* < .05, η _*p*_^2^ = 0.11, with older adults performing worse (*M* = 61.17) than young (*M* = 65.50). The age × emotion interaction, *F*(5, 230) = 2.00, *p* = .08, η _*p*_^2^ = 0.04, did not achieve significance, while the region × emotion interaction, *F*(10, 460) = 13.20, *p* < .001, η _*p*_^2^ = 0.22 and the interaction between region and age, *F*(2, 92) = 6.28, *p* < .01, η _*p*_^2^ = 0.12 were significant, supporting our hypothesis. There was also a strong three-way interaction between region, emotion, and age group, *F*(10, 460) = 6.55, *p* < .001, η _*p*_^2^ = 0.12, suggesting that age differences in perceiving emotions from different facial regions differed depending on the emotion (see Figure 1).

Bonferroni-corrected pairwise comparisons revealed that younger adults were significantly more accurate than older adults at perceiving *anger* (*p* = .031, *d* = 0.64) and *sadness* (*p* < .001, *d* = 1.16) from whole faces. Younger adults were also significantly better at perceiving *sadness* from the eyes than older adults (*p* = .001, *d* = 1.00). In direct contrast, older adults were found to be significantly better than younger adults at perceiving *anger* (*p* = .006, *d* = 0.83) and *sadness* (*p* = .018, *d* = 0.71) from the mouth region. However, younger adults were significantly more accurate than older adults at perceiving *fear* from the mouth (*p* = .002, *d* = 0.96). No other age effects were found to be significant (*p* = .141 to *p* = .996).

Extending the findings of Sullivan et al., (2007), older adults had particular difficulties (compared to young) in identifying sadness and anger from the whole face and sadness from eyes alone. In contrast, older adults were significantly better than younger at identifying sadness and anger from the mouth region, despite this being a very difficult task. As can be seen from Figure 1, this effect might be driven by younger participants having a larger drop in emotion perception accuracy for sad and angry mouths compared to eyes. This difference would be expected given the importance of the eyes for perceiving these emotions (Calder et al., 2000). However, the finding that older adults do not show an advantage in perceiving sadness and anger from the eyes compared to the mouth may indicate that they have difficulties decoding certain emotional information from the eyes. A different pattern was seen for fear, where there was a young-age advantage for mouths (but not eyes). How might this pattern of findings affect the decisions people make about emotions where information from eye and mouth regions is ambiguous or conflicting? This issue is addressed in Experiment 2.

## Experiment 2: Age Biases in Choosing Emotions From Chimeric Faces

Sometimes in social interactions the information presented by the eyes and mouth is not fully aligned: for example, when someone smiles politely despite being annoyed. One way to research this is to use chimeric expressions, which have different emotions portrayed in the eyes and mouth. Chimeric faces have traditionally been used to explore hemispheric dominance in emotion perception, displaying faces split vertically to show different emotions on the right and left (Prete et al., 2015). However, given previous findings that the upper region of the faces signals some emotions (e.g., anger, fear, and sadness), while the lower region is diagnostic of others (e.g., disgust and happiness; Calder et al., 2000; Eisenbarth & Alpers, 2011), then it would be profitable to explore chimeric faces when split horizontally across the upper–lower axis.

Prodan et al. (2007) presented cartoon line drawings of chimeric faces that displayed different emotions in upper and lower regions. They found that older adults had a greater bias toward choosing the emotion in the lower half of the faces. However, the study did not use real faces and presentation times were very brief (approx. 150 ms) and only to one side of the visual field. Crucially, data were not broken down by emotion.

Therefore, the main aim of Experiment 2 was to look at age differences in responses to photographs of emotional faces displaying incongruent emotions in the upper and lower regions. Given the findings from Experiment 1, it was predicted that older adults would be more likely than young to interpret a chimeric emotion in line with the expression shown in the mouth rather than the eyes, particularly where anger or sadness was portrayed. This was tested by calculating bias scores for each emotion which indicated the extent to which a participant tended to choose that emotion when it was shown in the eyes as opposed to the mouth.

### Method

#### Power analysis

A statistical power analysis was performed for sample size estimation, based on the meta-analytic data from Grainger and Henry (2020) on age differences in attention to the eyes, where the age-related *g* = 0.66, equivalent to *f* = 0.33. We therefore calculated the projected sample size needed to test our hypothesis of an interaction between age group and emotion influencing eye-bias score equivalent to this effect size. With alpha = 0.05 and power = 0.95 (G*Power 3.1, ANOVA repeated measures, age (2) as a between factor, assuming correlation between different conditions of 0.3, with a repeated measures factor of emotion * 6) suggests a minimum total sample size of *n* = 52. Our actual sample in this study of *n* = 80 should therefore be sufficient to detect the hypothesized age group effect. A sensitivity analysis with *n* = 80 revealed 95% power to detect a medium (*f* = 0.26) between participants factor.

#### Participants

Two groups of participants were recruited: 40 younger adults (eight males) aged 18–36 (*M* = 20.50, *SD* = 3.02) and 40 older adults (12 males) aged 65–81 (*M* = 70.93, *SD* = 4.30). They did not complete Experiment 1. Recruitment methods and criteria were the same as Experiment 1.

#### Stimuli

Photographs of eight actors (five females, three males) displaying each of the six basic emotions (anger, fear, sadness, happiness, surprise, and disgust) were selected from the FEEST (Young et al., 2002). All these images were of 100% emotional intensity. Therefore, there were 48 congruent stimuli in which the emotion displayed in the top and bottom half of the image matched. Incongruent chimeric stimuli were then created. In these images, two picture segments of different emotions portrayed by the same person were spliced together along the horizontal axis. The images were spliced in half across the middle of the nose, creating a chimeric face with one emotion being portrayed in the top half of the face and another displayed in the bottom half of the face. Combinations of emotions were determined using the Emotion Hexagon (Young et al., 2002), whereby chimeric faces were made by combining one emotion with the two emotions it was most likely to be mistaken for. There were 96 incongruent trials in total (6 emotions × 8 identities × 2 emotion combinations).

#### Procedure

Participants completed an emotion labeling task with 144 trials in total (48 congruent, 96 incongruent), which was split into two blocks of 72 trials. Congruent and incongruent trials were intermixed and presented in a randomized order: here we only analyze performance on the incongruent trials. Each face was presented individually in the center of a computer screen for 3 s, followed by the six emotion labels (anger, fear, sadness, happiness, disgust, and surprise). Participants were asked to decide which label best described the face shown in the trial, and made their response via a keypress. The next trial did not start until a response had been made.

We are primarily interested in the effects of aging on the bias to choose emotions from the upper or lower part of the chimeric faces. In order to explore this we calculated bias scores (following Prodan et al., 2007; Wegrzyn et al., 2017) by scoring +1 for any response which corresponded to the emotion in the upper part of the face and scoring −1 for any response corresponding to the emotion in the lower face. Responses which corresponded to any of the other four emotion labels were scored as 0. This results, for each emotion, in a bias score where positive values reflect a tendency to choose the emotion portrayed in the upper part of the face, and negative values a tendency to choose the emotion from the lower face.

### Results and Discussion

**Figure 2.**
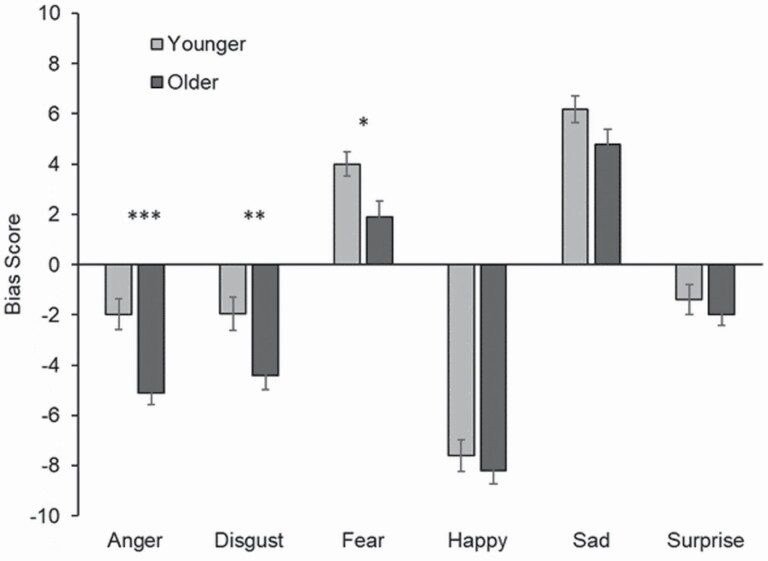
Experiment 2, bias scores broken down by emotion condition and age group. Positive score means more likely to choose emotion from eyes; negative scores suggest a bias for mouth. Error bars denote standard error.

To assess whether age of participant influenced bias toward choosing emotions from the mouth or the eyes, a 6 (emotion) × 2 (age group) mixed-design ANOVA was used. There were significant main effects of emotion, *F*(5, 390) = 226.39, *p* < .001, η _*p*_^2^ = 0.74, and age group, *F*(1, 78) = 9.71, *p* < .01, η _*p*_^2^ = 0.11, with older adults having more of a bias for choosing the mouth (*M* = −2.17) than younger adults (*M* = −0.46). There was also a significant emotion × age group interaction, *F*(5, 390) = 2.61, *p* < .05, η _*p*_^2^ = 0.032, suggesting that the tendency for older adults to choose the emotion shown in the mouth differed dependent on the emotion displayed. Bonferroni-corrected pairwise comparisons revealed that older adults had a greater bias toward using information from the lower part of the face to choose an anger (*p* < .001, *d* = 0.90) and disgust (*p* = .005, *d* = 0.64) label. When labeling fear, younger participants had a greater bias toward using information from the upper part of the face than older adults (*p* = .010, *d* = 0.59). There was no effect of age group for happiness (*p* = .465, *d* = 0.16), sadness (*p* = .090, *d* = 0.39), and surprise (*p* = .446, *d* = 0.17; see Figure 2).

When conflicting emotion information was presented in the eye and mouth regions of chimeric faces, older adults were more likely than young to use information from the mouth to make emotion decisions when anger, fear, or disgust were present. The same direction of age differences was seen for all emotions, but was only significant for these three. Taken together, this indicates that older adults are more able than young to identify anger from the mouth region (Experiment 1), and also more likely to use that information about the mouth in guiding decisions about anger (Experiment 2). Our final study explores the implications that age differences in biases for using emotional information from mouth have for decoding others’ emotions when the lower face region is covered with a face mask.

## Experiment 3: Effects of Face Masks Covering the Lower Half of the Face on Age Differences in Emotion Perception

In the current COVID-19 pandemic, wearing face masks that completely cover the lower half of the face has become widespread, and our results from Experiments 1 and 2 suggest that older adults may be particularly disadvantaged in terms of understanding emotions such as anger when face masks are worn and the mouth region is obscured. Indeed, it has been found that wearing of face masks in the COVID pandemic results in poorer emotion perception, particularly of sadness, anger, and fear (Carbon, 2020), and is likely to lead to miscommunication (Molnar-Szakacs et al., 2021). Face masks with a transparent window around the mouth have been designed to improve communication for those reliant on lip reading (Atcherson et al., 2017). Marini et al. (2021) show that such transparent mouth masks improve younger adults’ emotion perception compared to standard face masks. Given our evidence from Experiment 1 and 2 of the utility of the mouth region for older adults’ ability to identify emotions, we predict that transparent mouth masks will particularly assist older adults to make emotional judgments compared to standard masks. This may be particularly the case for emotions which older adults struggle most to identify from the eyes in Experiment 1 (anger and sadness).

It has been argued that hearing loss with age may play a role in directing attention away from the eyes and toward the mouth (Chaby et al., 2017). Hearing starts to decline between the ages of 30 and 40 (Mathers et al., 2000) and this might increasingly bias people toward the lower half of the face as lip reading becomes more important in discerning speech. Due to this increased attention to the mouth region over the life span, older adults may become more adept at decoding social cues from this region and, in turn, less likely to extract emotional information from the eyes. Here we directly assess the possible link between hearing and emotion perception from the eye region in old age.

In Experiment 3, we tested the hypothesis that older adults would be particularly impaired in emotion perception when the lower half of the face was occluded by a standard disposable face mask. We also tested whether older adults particularly benefited from the use of transparent-window “clear mouth masks.” As in the previous studies, we predicted that the age effects of face masks would be greatest for anger and sadness. We also assessed the relationship between hearing perception and emotion perception performance in the standard face mask condition.

### Method

#### Power analysis

Here our primary hypothesis is of an interaction between age group and face mask type on emotion perception. We have no previous studies on which to base a clear estimate of the potential size of this interaction. Given the results of the previous two experiments we predicted a medium effect size, *f* = 0.25. Therefore, a statistical power analysis was performed for sample size estimation, to test our hypothesis of an interaction between age group (2) and mask type (3). With alpha = 0.05 and power = 0.95 (G*Power 3.1, ANOVA repeated measures, within–between interaction, assuming correlation between different conditions of 0.3) suggests a minimum total sample size of *n* = 60. Our actual sample of *n* = 170 should therefore be sufficient to detect the hypothesized interaction. Note that we substantially overrecruited for this study because it was an online study, so we expected to find more variability in the data. A sensitivity analysis revealed 95% power to detect a small to medium between–within interaction effect (*f* = 0.15).

#### Participants

Eighty-two younger adults (eight males) aged 17–30 (*M* = 20.71, *SD* = 2.44) and 88 older adults (36 males) aged 60–84 (*M* = 68.48, *SD* = 6.04) completed this online study. They did not complete the previous experiments. Participants were recruited through local pools (see Experiment 1), with additional participants recruited through Prolific Academic.

#### Stimuli

**Figure 3.**
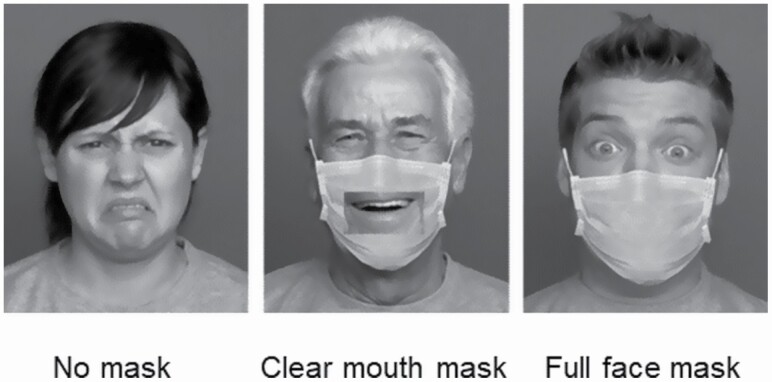
Sample stimuli used in Experiment 3. In Experiment 3, we tested whether older adults were particularly impaired in emotion perception when the lower half of the face was obscured with a face mask, and whether a transparent mouth mask was particularly beneficial for older adults. In the examples shown: the no mask condition depicts disgust; the clear mouth mask, happiness; and the full mask, fear. Stimuli from Experiment 3 were adapted from the FACES database described by Ebner et al. (2010) and were presented in color during the task.

Color pictures of younger and older actors portraying facial expressions were retrieved from the FACES database (Ebner et al., 2010). Images of 16 individuals (four older females, four older males, four younger females, four younger males) portraying each of six facial expressions (anger, disgust, fear, happiness, neutral, and sadness: note that this database does not include surprise) were selected and edited using the GNU Image Manipulation Program to add in a face covering: either a standard disposable face mask, or a transparent-window “clear mouth” mask; see Figure 3 for examples.

#### Procedure

Participants accessed the experiment (written in Testable) online using a computer or laptop, with a calibration check to scale the stimuli with the resolution of each participant’s screen. After providing demographic information, participants completed one block of six practice trials, followed by eight blocks of 36 (a total of 288) self-paced experimental trials. On each trial, a photograph of a face was presented (no face mask, clear mouth mask, full face mask) along with the six possible emotion labels (anger, disgust, fear, happiness, neutral, and sadness), and participants were asked to identify the displayed emotional expression by clicking on the label that they thought best described the emotion depicted. The presentation order of stimuli was randomized across the sample. As the data were collected online we checked each participant’s data set for any sign of unusual patterns. Two older participants showed evidence of never using one of the emotion labels to make responses, so they were excluded from the sample described above.

After completing the emotion categorization task, participants were asked to complete a second session involving some questionnaires. Sixty-six younger and 84 older participants completed the 25-item Hearing Handicap Inventory in the Elderly (HHIE) Screening Questionnaire to assess individual differences in hearing ability (Ventry & Weinstein, 1982), where larger scores indicate more hearing difficulties.

### Results and Discussion

To investigate the effects of age (younger, older adults), face mask condition (no face mask, clear mouth mask, full face mask), and emotion (anger, disgust, fear, happiness, neutral, sadness), a mixed-design ANOVA was conducted. There were significant main effects of emotion, *F*(5, 840) = 214.35, *p* < .001, η _*p*_^2^ = 0.561 and age group, *F*(1, 168) = 20.16, *p* < .001, η _*p*_^2^ = 0.107 with older adults (*M* = 81.50) being significantly less accurate than young (*M* = 85.61). Face mask condition influenced performance, *F*(2, 336) = 333.96, *p* < .001, η _*p*_^2^ = 0.665 with significantly worse performance in full (*M* = 77.59) compared to clear mouth mask (*M* = 85.36) conditions, both being performed worse than the no mask condition (*M* = 87.51).

**Figure 4.**
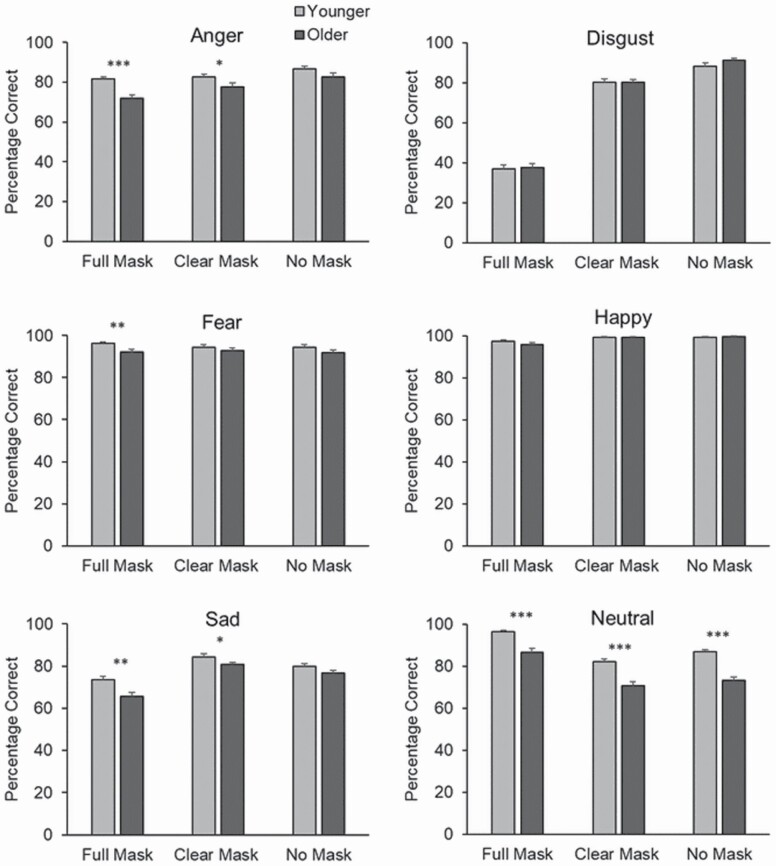
Experiment 3, mean percentage correct for each Emotion category broken down by face mask condition and age group. Error bars denote standard error.

There were significant two-way interactions between face mask condition and age group, *F*(2, 336) = 3.621, *p* < 0.05, η _*p*_^2^ = 0.021, age group and emotional expression, *F*(5, 840) = 9.54, *p* < .001, η _*p*_^2^ = 0.054, and face mask condition and emotional expression, *F*(10, 1680) = 374.16, *p* < .001, η _*p*_^2^ = 0.690. However, these two-way interactions were qualified by a significant three-way interaction between age, face mask condition, and emotion, *F*(10, 1680) = 2.08, *p* < .05, η _*p*_^2^ = 0.012; see Figure 4.

Bonferroni-corrected pairwise comparisons for each emotion revealed that older adults were considerably worse at identifying *anger* from the full mask condition (*p* < .001, *d* = 0.65), while the age difference was considerably reduced in the clear mask condition (*p* = .031, *d* = 0.33), and nonsignificant in the no mask condition (*p* = .086, *d* = 0.28). For *sadness*, older adults were much worse than younger adults in the full mask (*p* = .004, *d* = 0.45), the age difference was reduced in the clear mask condition (*p* = .046, *d* = 0.31), but there were no age differences in the no mask condition (*p* = .128, *d* = 0.24). There was a significant age-related difference in perceiving *fear* in the full mask condition (*p* = .004, *d* = 0.45) but this effect was not found in the clear mask (*p* = .296, *d* = 0.16) and no mask condition (*p* = .149, *d* = 0.22). There were no age differences found in any mask condition for *happiness* or *disgust* (*p* from .086 to .997). Finally, for *neutral* faces older adults performed worse than younger participants across all conditions (full mask *p* < .001, *d* = 0.76, clear mask *p* < .001, *d* = 0.79, no mask *p* < .001, *d* = 1.00).

Final analyses explored the relationship between hearing ability and emotion perception. As expected, younger adults (*M* = 3.89, *SD* = 6.68) reported having fewer problems with hearing than older adults (*M* = 8.19, *SD* = 13.30), *t*(150) = 2.42, *p* < .05, *d* = 0.41. However, there were no significant correlations between HHIE scores and emotion perception in the full face mask condition in either younger *r*(64) = −0.096, *p* = .44 or older adults *r*(84) = −0.123, *p* = .26.

Face masks impaired emotion perception overall (supporting Carbon, 2020) and (collapsing across emotions) the aging effect size was similar for no mask (*d* = 0.54) and clear mouth mask (*d* = 0.58) conditions, and much larger for the full mask condition (*d* = 0.76). This suggests that older adults were particularly impaired when trying to decode emotions without access to information from the lower half of the face, and this was ameliorated when a clear mouth mask was used instead. We extended this to look at age effects in identifying specific emotions. Full face masks caused older adults to become much worse than young at labeling anger, sadness, and fear. Age effects were smaller or nonsignificant when identifying these emotions from a clear mask or no mask, indicating the utility of clear masks in assisting older adults. There were no age differences in identifying happiness or disgust, and older adults’ poorer ability to identify neutral faces was equivalent across mask conditions.

Older adults rated themselves as having more hearing difficulties than young. However, there was no association between self-rated hearing loss and emotion perception performance in the full face mask condition, suggesting that age-related difficulties in using emotional information from the eyes are not related to hearing problems.

## General Discussion

The main aim of the current research was to assess age-related differences in decoding emotions from the eyes and the mouth. In line with predictions, older adults were worse than young at perceiving anger and sadness from photographs of the eye region, but these age effects were reversed in the difficult task of identifying emotions from the mouth region (Experiment 1). Only for fear did older adults’ difficulty in perceiving emotion extend to the lower half of the face. Further, age-related biases away from using information from the eye region were found when making judgments of chimeric faces in which the emotions shown in the eye and mouth region conflicted (Experiment 2). Specifically, older adults were more biased than young to choose anger, fear, and disgust when shown in the lower part of the face. In Experiment 3, it was found that older adults were disproportionately impaired at perceiving anger, sadness, and fear when the mouth region was covered with a face mask. Therefore, the current research suggests that older adults have difficulties interpreting emotions, particularly anger and sadness, from the eye region alone and they also find this region less salient when making holistic judgments of emotional faces. These results have implications for understanding the nature of both age differences in emotion perception (Hayes et al., 2020) and social attention to different facial regions (Grainger & Henry, 2020).

Previous research argued that age-related differences in emotion perception might be due to older adults paying less attention to the eye region (Wong et al., 2005). However, in Experiment 1, there was evidence of age-related declines in the ability to perceive emotions when *only* the eye region of the face was presented so older adults would have had to look at this region when making their decision. This suggests that rather than reduced attention to the eyes influencing emotion perception in old age, older adults seem to have problems extracting and decoding the relevant emotional information from this region. This suggestion is in keeping with previous visual scanning studies which have found no association between fixations to the eyes and emotion perception performance in older adults (Grainger et al., 2017; Murphy & Isaacowitz, 2009; Sullivan et al., 2007).

Instead, the findings of the current experiments also contribute to the growing body of literature indicating that older adults have difficulties processing information from the eyes. For example, there is evidence of age-related declines in the ability to decode complex mental states from the eye region (Bailey et al., 2008). Also, older adults have difficulties detecting subtle differences in eye-gaze direction and using these social cues to engage in joint attention with others (Slessor et al., 2008) and as a cue to deceit (Slessor et al., 2012). Therefore, older adults might be biased toward using cues from the mouth of emotion faces as they are more adept at using this information.

Why do older adults rely more on the mouth region of the face (and less on the eyes) when decoding emotional expressions? One previous suggestion was that age-related hearing loss might cause older adults to focus on the mouth of faces in order to lip read (Chaby et al., 2017). This might lead to more adept processing of that region and less focus on decoding emotion cues from the eyes. In Experiment 3, we directly tested this possible link but found that there was no significant relationship between self-assessed hearing ability and emotion perception from eyes. In addition, a lip-reading explanation does not explain why older adults would only show a significant mouth bias for some emotional expressions. These findings argue against a lip-reading expertise explanation for the relative ability of older adults to discern emotions from mouths compared to eyes. However, we used a self-rated assessment of hearing difficulties and it would be useful in future research to use more detailed and objective audiometric measurement.

Another possibility is that age-related declines in the ability to decode emotional information from the eye region might relate to declining visual perception with age. Age-related declines in both visual acuity and contrast sensitivity are well documented (Gittings & Forzard, 1986; Greene & Madden, 1987). The eyes cover a relatively small area of the face and making judgments from this region often involves fine-grained and subtle distinctions. In contrast, visual cues from mouths may be larger and easier to discern. Again, it is unlikely that this explanation could account for age-related mouth biases being specific to certain emotions. However, future research should carry out detailed assessment of visual function to understand the role of perceptual factors in age differences in discerning emotions from different facial regions.

Age effects were not uniform across emotions, and were most consistent for the negative emotions of anger and sadness. Sullivan et al. (2007) and Ebner et al. (2011) argued that older adults might be from a generation that considers it socially unacceptable to look others directly in the eye and thus is more likely to avoid eye contact during social interactions. This could mean that, over time, older adults have become more attuned to picking up social cues from the mouth rather than the eyes. This might be particularly evident for negative emotions such as anger and sadness due to older adult’s having a positivity bias to avoid negative or threatening emotions that are directed toward them (Mather & Carstensen, 2003). In order to explore these different interpretations future research should assess whether attitudes to eye contact and age-related positivity biases correlate with emotion perception from the eyes and mouth region.

It is also important to note that the effects of age on the perception of specific emotions from the whole face were not consistent across experiments. For example, in Experiment 1, older adults were significantly worse at perceiving anger and sadness in the full face condition, while in the no mask condition in Experiment 3, significant age-related differences were only found for neutral faces. This discrepancy might be due to a number of methodological differences between the two experiments. For example, neutral images and a neutral response option were included in Experiment 3 and surprised facial expressions were not included, which could have led to different biases in responding. In addition, the inclusion of images with masks in the trial blocks may have altered processing strategies. Future research should further explore how these methodological factors could influence age-related differences in the perception of emotional facial expressions.

In both age groups, the relative weight given to mouths and eyes in guiding the processing of different emotions roughly fits with previous literature (e.g., Calder et al., 2000). In other words, emotions such as happiness and disgust were more likely to be identified by the emotion shown in the mouth, while fear and sadness were more likely to be associated with the eyes. Perhaps more surprising was the pattern for anger, where in Experiment 2, both younger and older adults tended to label faces as angry more from the lower than upper half of the face. Anger stereotypically involves the display of both a furrowed brow and an open mouth with bared teeth, so logically either cue might be important. In fact, Wegrzyn et al. (2017, see their Figure 5) show that two different angry faces varied a lot in the extent to which upper and lower face regions helped to classify the emotion.

The current research is limited by using controlled but artificial stimuli which lack ecological validity (Kunzmann & Isaacowitz, 2017). We only used static photographs of faces, but there is evidence that older adults can benefit from dynamic stimuli in emotion perception tasks (Grainger et al., 2017). It could be argued that older adults might have been particularly disadvantaged in Experiments 1 and 2 as the age of the face was not systematically varied and stimuli tended to be of younger adults. However, previous research has indicated that there is no evidence for an own-age bias in emotion perception and, if anything, both age groups are worse at perceiving emotional expressions of older adults (Fölster et al., 2014). Only traditional “basic” emotions were explored, because they are the best understood in relation to patterns of processing the eyes and mouth, but it is also important to explore these age effects in relation to more complex, fleeting, and nuanced emotions. Given the importance of providing contextual information for interpreting emotions, particularly for older adults (Ngo & Isaacowitz, 2015; Noh & Isaacowitz, 2013), future studies should also look at the role of eye and mouth cues to emotion in contextualized settings such as in health care, particularly as there is evidence that the use of face masks reduces empathy in doctor–patient consultations (Wong et al., 2013).

A further limitation of the present research was that older participants were not screened for mild cognitive impairment or dementia. All older adults who participated lived at home independently and were actively engaged in research. There was no evidence of any outliers in the data. However, future research should investigate the effects of mild cognitive impairment and dementia on eye and mouth biases in emotion perception.

In Experiment 3, we only explored age-related differences in emotion perception when the mouth was covered, due to the recent increase in mask wearing throughout the COVID-19 pandemic. However, there are also situations where the eye region is occluded. For example, when someone is wearing glasses or sunglasses. Noyes et al. (2021) found that participants’ ability to perceive anger, sadness, and fear was impaired when the face image was wearing sunglasses. Future research exploring whether glasses and sunglasses can influence older adults’ perception of these emotions would make an important contribution to the literature.

Given the ongoing COVID-19 pandemic and a related increase in awareness of disease transmission, it is likely that the wearing of face masks that obscure the bottom half of the face will be widespread for some time. Therefore, findings that the disruption in ability to perceive facial expressions of anger, fear, and sadness was greatest for older adults could have important implications for social communication generally and also more specifically in health care settings where mask wearing is mandatory. However, extending the findings of Marini et al. (2021), a mask with a transparent region around the mouth considerably improved older adults’ perception of emotional expressions. Therefore, altering the design of face masks could have important consequences for improving communication for older people.

In sum, older adults were less accurate than young at identifying emotions such as anger and sadness from the eye region, less likely to use information from the eyes when judging conflicting emotions but better than young at identifying anger and sadness from the mouth region. As a consequence of relying more on lower face information when identifying emotions, older adults had particular difficulties perceiving anger, sadness, and fear when the bottom half of the face was fully covered with a face mask. However, they showed improved emotion perception when the mask had a transparent window revealing the mouth region of the face. These findings have important theoretical implications for understanding age differences in social attention and practical implications for face mask design.

## Data Availability

Data, analytic methods, and study materials from Experiment 3 will be made available to other researchers. To request access to these, please e-mail louise.phillips@abdn.ac.uk. The study materials used in Experiment 1 and 2 are subject to copyright and therefore we cannot share these. The experiments in this manuscript were not preregistered.

## References

[CIT0001] Atcherson, S. R., Mendel, L. L., Baltimore, W. J., Patro, C., Lee, S., Pousson, M., & Spann, M. J. (2017). The effect of conventional and transparent surgical masks on speech understanding in individuals with and without hearing loss. Journal of the American Academy of Audiology, 28(1), 58–67. doi:10.3766/jaaa.15151 28054912

[CIT0002] Bailey, P. E., Henry, J. D., & von Hippel, W. (2008). Empathy and social functioning in late adulthood. Aging and Mental Health, 12(4), 499–503. doi:10.1080/13607860802224243 18791898

[CIT0004] Birmingham, E., Svärd, J., Kanan, C., & Fischer, H. (2018). Exploring emotional expression recognition in aging adults using the Moving Window Technique. PLoS ONE, 13(10), Article e0205341. doi:10.1371/journal.pone.0205341 30335767 PMC6193651

[CIT0005] Calder, A. J., Young, A. W., Keane, J., & Dean, M. (2000). Configural information in facial expression perception. Journal of Experimental Psychology Human Perception and Performance, 26(2), 527–551. doi:10.1037//0096-1523.26.2.527 10811161

[CIT0006] Carbon, C . (2020). Wearing face masks strongly confuses counterparts in reading emotions. Frontiers in Psychology, 11. doi:10.3389/fpsyg.2020.566886 PMC7545827 33101135

[CIT0007] Chaby, L., Hupont, I., Avril, M., Luherne-du Boullay, V., & Chetouani, M. (2017). Gaze behavior consistency among older and younger adults when looking at emotional faces. Frontiers in Psychology, 8, 548. doi:10.3389/fpsyg.2017.00548 28450841 PMC5390044

[CIT0009] Ebner, N. C., He, Y., & Johnson, M. K. (2011). Age and emotion affect how we look at a face: Visual scan patterns differ for own-age versus other-age emotional faces. Cognition & Emotion, 25(6), 983–997. doi:10.1080/02699931.2010.540817 21614704 PMC3339265

[CIT0010] Ebner, N. C., Riediger, M., & Lindenberger, U. (2010). FACES—A database of facial expressions in young, middle-aged, and older men and women: Development and validation. Behavior Research Methods, 42(1), 351–362. doi:10.3758/BRM.42.1.351 20160315

[CIT0011] Eisenbarth, H., & Alpers, G. W. (2011). Happy mouth and sad eyes: Scanning emotional facial expressions. Emotion, 11(4), 860–865. doi:10.1037/a0022758 21859204

[CIT0012] Fölster, M., Hess, U., & Werheid, K. (2014). Facial age affects emotional expression decoding. Frontiers in Psychology, 5, 30. doi:10.3389/fpsyg.2014.00030 24550859 PMC3912746

[CIT0013] Gittings, N. S., & Forzard, J. L. (1986). Age related changes in visual acuity. Experimental Gerontology, 21(4–5), 423–433. doi:10.1016/0531-5565(86)90047-1 3493168

[CIT0014] Grainger, S. A., & Henry, J. D. (2020). Gaze patterns to emotional faces throughout the adult lifespan. Psychology and Aging, 35(7), 981–992. doi:10.1037/pag0000571 32816505

[CIT0015] Grainger, S. A., Henry, J. D., Phillips, L. H., Vanman, E. J., & Allen, R. (2017). Age deficits in facial affect recognition: The influence of dynamic cues. The Journals of Gerontology, Series B: Psychological Sciences and Social Sciences, 72(4), 622–632. doi:10.1093/geronb/gbv100 26530079

[CIT0016] Greene, H. A., & Madden, D. J. (1987). Adult age differences in visual acuity, stereopsis, and contrast sensitivity. American Journal of Optometry and Physiological Optics, 64(10), 749–753. doi:10.1097/00006324-198710000-00006 3688177

[CIT0017] Hayes, G. S., MacLennan, S. N., Henry, J. D., Phillips, L. H., Terret, G., Rendell, P. G., Pelly, R. M., & Labuschagne, I. (2020). Task characteristics influence facial emotion recognition age-effects: A meta-analytic review. Psychology and Aging, 35(2), 295–315. doi:10.1037/pag0000441 31999152

[CIT0018] Kunzmann, U., & Isaacowitz, D. (2017). Emotional aging: Taking the immediate context seriously. Research in Human Development, 14(3), 182–199. doi:10.1080/15427609.2017.1340048

[CIT0019] Mather, M., & Carstensen, L. L. (2003). Aging and attentional biases for emotional faces. Psychological Science, 14(5), 409–415. doi:10.1111/1467-9280.01455 12930469

[CIT0020] Mathers, C., Smith, A. W., & Concha, M. (2000). Global burden of hearing loss in the year 2000. Global Burden of Disease, 18(4), 1–30.

[CIT0021] Marini, M., Ansani, A., Paglieri, F., Caruana, F., & Viola, M. (2021). The impact of facemasks on emotion recognition, trust attribution and re-identification. Scientific Reports, 11(5577). doi:10.1038/s41598-021-84806-5 PMC7970937 33692417

[CIT0022] Molnar-Szakacs, I., Uddin, L. Q., & Heffernan, M. B. (2021). The face behind the mask: The future of interpersonal interaction. Neuron, 109(12), 1918–1920. doi:10.1016/j.neuron.2021.05.030 34139182 PMC8730492

[CIT0023] Montagne, B., Kessels, R. P. C., DeHaan, H. E. F., & Perrett, D. I. (2007). The emotion recognition task: A paradigm to measure the perception of facial emotional expressions at different intensities. Perceptual and Motor Skills, 104(2), 589–598. doi:10.2466/PMS.104.2.589-598 17566449

[CIT0024] Murphy, N. A., & Isaacowitz, D. M. (2009). Age effects and gaze patterns in recognising emotional expressions: An in-depth look at gaze measures and covariates. Cognition and Emotion, 24(3), 436–452. doi:10.1080/02699930802664623

[CIT0025] Ngo, N., & Isaacowitz, D. M. (2015). Use of context in emotion perception: The role of top-down control, cue type, and perceiver’s age. Emotion, 15(3), 292–302. doi:10.1037/emo0000062 25985276

[CIT0026] Noh, S. R., & Isaacowitz, D. M. (2013). Emotional faces in context: Age differences in recognition accuracy and scanning patterns. Emotion, 13(2), 238–249. doi:10.1037/a0030234 23163713 PMC4119600

[CIT0027] Noyes, E., Davis, J. P., Petrov, N., Gray, K. L., & Ritchie, K. L. (2021). The effect of face masks and sunglasses on identity and expression recognition with super-recognizers and typical observers. Royal Society Open Science, 8(3), 201169. doi:10.1098/rsos.201169 33959312 PMC8074904

[CIT0028] Prete, G., Laeng, B., Fabri, M., Foschi, N., & Tommasi, L. (2015). Right hemisphere or valence hypothesis, or both? The processing of hybrid faces in the intact and callosotomised brain. Neuropsychologia, 68, 94–106. doi:10.1016/j.neuropsychologia.2015.01.002 25575451

[CIT0029] Prodan, C. I., Orbelo, D. M., & Ross, E. D. (2007). Processing of facial blends of emotion: Support for right hemisphere cognitive aging. Cortex: A Journal Devoted to the Study of the Nervous System and Behavior, 43(2), 196–206. doi:10.1016/S0010-9452(08)70475-1 17405666

[CIT0030] Slessor, G., Phillips, L. H., & Bull, R. (2008). Age-related declines in basic social perception: Evidence from tasks assessing eye-gaze processing. Psychology and Aging, 23(4), 812–822. doi:10.1037/a0014348 19140652

[CIT0031] Slessor, G., Phillips, L. H., Bull, R., Venturini, C., Bonny, E., & Rokaszewicz, A. (2012). Investigating the “deceiver stereotype”: Do older adults associate averted gaze with deception? The Journals of Gerontology, Series B: Psychological Sciences and Social Sciences, 67(2), 178–183. doi:10.1093/geronb/gbr087 21808070

[CIT0032] Sullivan, S., Ruffman, T., & Hutton, S. B. (2007). Age differences in emotion recognition skills and visual scanning on emotion faces. The Journals of Gerontology, Series B: Psychological Sciences and Social Sciences, 62(1), 53–60. doi:10.1093/geronb/62.1.P53 17284558

[CIT0033] Ventry, I. M., & Weinstein, B. E. (1982). The Hearing Handicap Inventory for the Elderly: A new tool. Ear and Hearing, 3(3), 128–134. doi:10.1097/00003446-198205000-00006 7095321

[CIT0034] Wegrzyn, M., Vogt, M., Kireclioglu, B., Schneider, J., & Kissler, J. (2017). Mapping the emotional face. How individual face parts contribute to successful emotion recognition. PLoS One, 12(5), e0177239. doi:10.1371/journal.pone.0177239 28493921 PMC5426715

[CIT0035] Wong, B., Cronin-Golomb, A., & Neargarder, S. (2005). Patterns of visual scanning as predictors of emotion identification in normal aging. Neuropsychology, 19(6), 739–749. doi:10.1037/0894-4105.19.6.739 16351349

[CIT0036] Wong, C. K. M., Yip, B. H. K., Mercer, S., Griffiths, S., Kung, K., Wong, M. C., Chor, J., & Wong, S. Y. (2013). Effect of facemasks on empathy and relational continuity: A randomised controlled trial in primary care. BMC Family Practice, 14, 200. doi:10.1186/1471-2296-14-200 24364989 PMC3879648

[CIT0037] Young, A., Perrett, D., Calder, A., Sprengelmeyer, R., & Ekman, P. (2002). Facial Expressions of Emotion: Statistics and Tests (FEEST) manual. Thames Valley Test Company.

